# Toward Automated Data Extraction According to Tabular Data Structure: Cross-sectional Pilot Survey of the Comparative Clinical Literature

**DOI:** 10.2196/33124

**Published:** 2021-11-24

**Authors:** Karl Holub, Nicole Hardy, Kevin Kallmes

**Affiliations:** 1 Nested Knowledge St. Paul, MN United States

**Keywords:** table structure, systematic review, automated data extraction, data reporting conventions, clinical comparative data, data elements, statistic formats

## Abstract

**Background:**

Systematic reviews depend on time-consuming extraction of data from the PDFs of underlying studies. To date, automation efforts have focused on extracting data from the text, and no approach has yet succeeded in fully automating ingestion of quantitative evidence. However, the majority of relevant data is generally presented in tables, and the tabular structure is more amenable to automated extraction than free text.

**Objective:**

The purpose of this study was to classify the structure and format of descriptive statistics reported in tables in the comparative medical literature.

**Methods:**

We sampled 100 published randomized controlled trials from 2019 based on a search in PubMed; these results were imported to the AutoLit platform. Studies were excluded if they were nonclinical, noncomparative, not in English, protocols, or not available in full text. In AutoLit, tables reporting baseline or outcome data in all studies were characterized based on reporting practices. Measurement context, meaning the structure in which the interventions of interest, patient arm breakdown, measurement time points, and data element descriptions were presented, was classified based on the number of contextual pieces and metadata reported. The statistic formats for reported metrics (specific instances of reporting of data elements) were then classified by location and broken down into reporting strategies for continuous, dichotomous, and categorical metrics.

**Results:**

We included 78 of 100 sampled studies, one of which (1.3%) did not report data elements in tables. The remaining 77 studies reported baseline and outcome data in 174 tables, and 96% (69/72) of these tables broke down reporting by patient arms. Fifteen structures were found for the reporting of measurement context, which were broadly grouped into: 1×1 contexts, where two pieces of context are reported in total (eg, arms in columns, data elements in rows); 2×1 contexts, where two pieces of context are given on row headers (eg, time points in columns, arms nested in data elements on rows); and 1×2 contexts, where two pieces of context are given on column headers. The 1×1 contexts were present in 57% of tables (99/174), compared to 20% (34/174) for 2×1 contexts and 15% (26/174) for 1×2 contexts; the remaining 8% (15/174) used unique/other stratification methods. Statistic formats were reported in the headers or descriptions of 84% (65/74) of studies.

**Conclusions:**

In this cross-sectional pilot review, we found a high density of information in tables, but with major heterogeneity in presentation of measurement context. The highest-density studies reported both baseline and outcome measures in tables, with arm-level breakout, intervention labels, and arm sizes present, and reported both the statistic formats and units. The measurement context formats presented here, broadly classified into three classes that cover 92% (71/78) of studies, form a basis for understanding the frequency of different reporting styles, supporting automated detection of the data format for extraction of metrics.

## Introduction

### Extracting Data for a Systematic Review

Systematic reviews and meta-analyses of high-quality studies are essential for clinical decision-making [1], guidelines [2], and evidence-based adoption and approval of therapies [3]. Quantitative data extraction is an essential task in the systematic review/meta-analysis process, during which researchers gather patient characteristics, interventions, and outcomes of interest in a common format to support summarization and statistical analysis. The current practice for data extraction is manual review of published manuscripts of studies, with subsequent manual entry of data into a spreadsheet or review software [4]. The manual, work-intensive nature of this task contributes to the high cost in time and money of reviewing the clinical literature.

The time investment and costs of systematic reviews/meta-analyses—which can reach 16 months and US $141,000 [5] in labor to complete a single review—are the major limiting factors in the synthesis of scientific evidence. The task of data extraction from published comparative studies typically demands 20% of the total review and analysis time, and is subject to high accuracy standards [6,7]. This has led to calls for both improved software systems for systematic reviews/meta-analyses and automation of the data extraction process. However, according to a systematic review of systematic review/meta-analysis extraction automation projects, “no unified information extraction framework [has been] tailored to the systematic review process…[automation] techniques have not been fully utilized to fully or even partially automate the data extraction step of systematic review” [8].

### Systematic Review Workflow Software Platforms

Despite the fact that automated data extraction for systematic reviews/meta-analyses has yet to be achieved, several web-based software options currently support part or all of the workflow of a review [9], establishing a systematic approach on which automated data extraction can be modeled. We previously developed a workflow software platform (AutoLit, Nested Knowledge, MN) [10] for performing and presenting systematic reviews and meta-analyses. The data extraction functions of AutoLit are user-driven and focused on extracting descriptive statistics. After articles are retrieved and screened, users read the PDFs of study content and feed extracted data directly into a database, which is used to produce a “living” summary and obtain interpretive statistical outputs. This platform has provided a basis for experimentation with the streamlining of data extraction, the end goal of which is automated identification, parsing, and abstraction of summary statistics reported in medical manuscripts.

### Automated Data Extraction Efforts

To begin to solve the problem of automated data extraction, it is first essential to understand the format in which input data is available. PDF manuscripts are the de facto publication medium. Within these manuscripts, the key data regarding the patient population/characteristics, interventions of interest, and outcomes are presented in both the text and data tables. Notably, the majority of previous extraction efforts have focused on textual extraction [8], despite the varied presentation styles and unstructured nature of both contextual information and the data themselves.

### Targeting Extraction from Tables

We hypothesized that data tables, as opposed to free text, represent an ideal target for automated extraction based on the following traits: (1) data in tables are densely concentrated; (2) tables are delimited and typically include structure and a standardized set of contexts not found in free text; (3) tables often report statistics not mentioned in the free text (eg, secondary outcomes); and tables consistently report data with higher precision and full information (eg, dispersion measures and sample sizes).

### Existing Standards for Tabular Presentation

Journals and medical research bodies have published standards related to how statistics and tables should be presented [11-13]. Common themes include presenting units for continuous data elements, standardization of statistic formatting (eg, mean [SD]), reporting interventions as full names or standardized abbreviations, and reporting sample sizes used in an analysis.

Despite these guidelines, table formatting standards, both in terms of style and content, vary between journals. This heterogeneity has been noted previously, and a software tool targeted toward authors [14], “tableone,” was created to enable harmonized generation of statistical analyses and tables directly from study results. However, tableone and similar tools are not yet widely adopted to a sufficient extent to meaningfully standardize reporting.

### Classifying Tabular Reporting Practices

Given this context, automated tabular extraction depends on understanding the variety of table structures—including the types and frequencies of variation from common formats—in medical manuscripts. A recent systematic review showed that although 14 independent automation projects focused on full-text extraction have been published [15], only one project focused on extraction from tables [8]. Furthermore, although the results of this project were promising, achieving high accuracy in machine learning–based extraction, neither this nor any other study to date has surveyed or classified the structure of tables presented in the clinical literature. Therefore, in this study, we focused on identifying the characteristics that are essential for the automation of extraction of descriptive statistics in tables. This can provide concrete structural characteristics to enable assessment of the generalizability of tabular presentation formats and support the future automation of extraction.

## Methods

### Sampling Published Comparative Clinical Studies

A cross-sectional sample of clinical study publication records was generated. In brief, published studies tagged as randomized controlled trials (RCTs), as indexed in PubMed, from 2019 were searched using the following term: “randomized controlled trial” [Publication Type] AND 2019/01/01:2020/01/01[dp]. Search results were exported from PubMed on August 9, 2021, using the Entrez application programming interface [16]; imported to the AutoLit platform; and search results were randomly sampled by index, without replacement, using the R function “sample” to select 100 records. Of these, all published articles were included except those that met the following exclusion criteria: not clinical, not comparative (ie, the publication does not compare outcomes between patient groups), not in English, protocol only, or no full text available.

### Abstracting Tables

Within this sample, table attributes were identified, classified, and summarized. The concepts for data extraction used are summarized in Table 1.

**Table 1:** Defining tabular concepts.

Concept	Definition	Example
Data element	A characteristic or quality being measured	Mortality
Metric	A measured instance of a data element, a descriptive statistic	2/59 (3.4%)
Arm	A subset of an experiment’s participants that are assigned a specific intervention	Placebo group
Time point	The point(s) in time in an experiment when measurement of data elements is performed	6-month follow-up
Measurement context	The combination of a data element, arm, and time point in experimental reporting	Mortality in the placebo group at 6 months

Data extraction necessarily has to address the attributes of measurement context to discern the meaning of a given metric. Thus, for each patient arm, the intervention and population size must be identified, and for each data element, the unit of measurement and time points must be extracted. Furthermore, metrics must be parsed into their constituent statistics, including (1) continuous metrics as the measure of central tendency and the dispersion measure; (2) dichotomous metrics, namely the subset, total population, and percentage (n/N, %); and (3) categorical metrics, namely the subset and total population.

**Figure 1:**
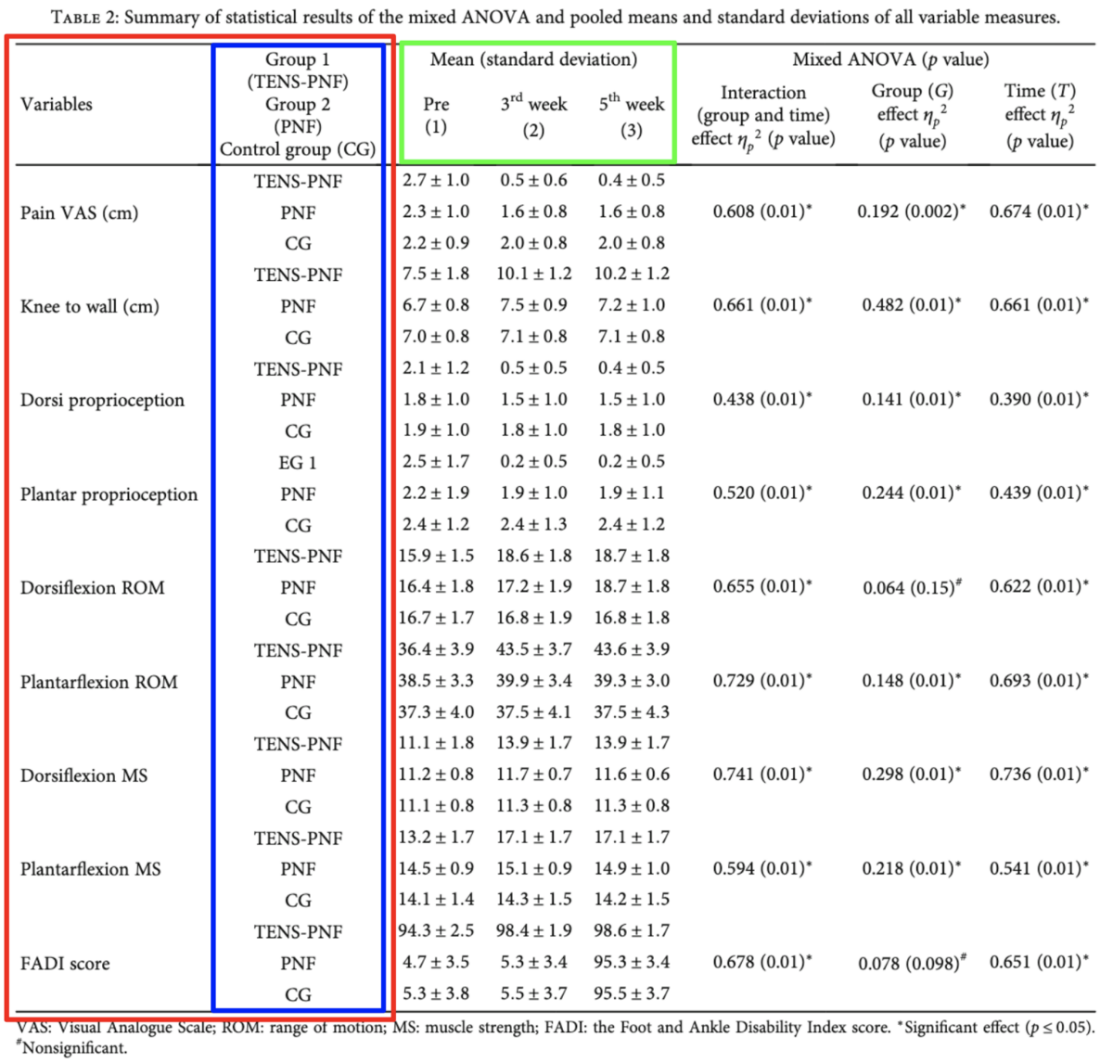
Example of a 2x1 context with arms (blue) nested in data elements (red) on rows and time points (green) on the columns. Table from Chellappa et al [17]. ANOVA: analysis of variance.

**Figure 2:**
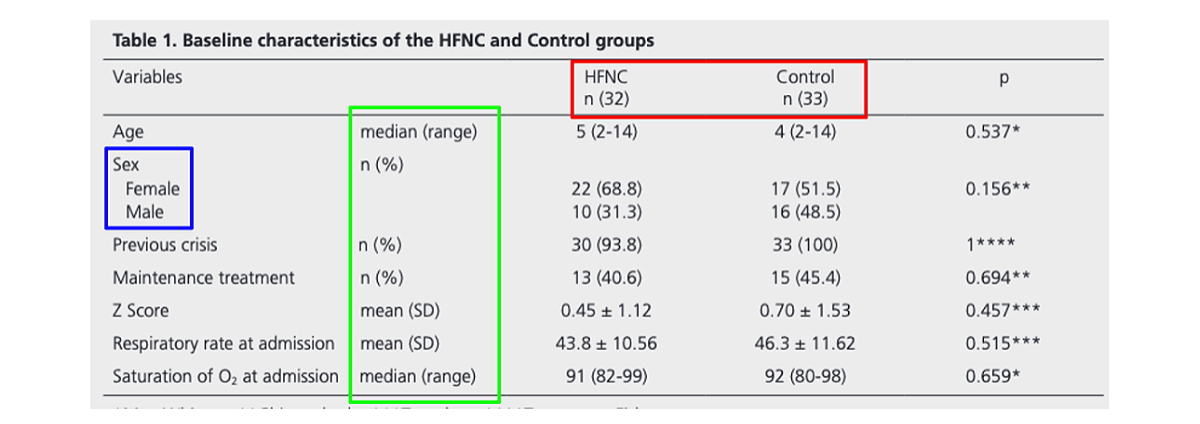
A 1x1 baseline table reporting data elements on rows and arms on columns. Arm sizes are embedded in intervention headers (red), category labels are reported in the data element array indented (blue), and statistic formats are reported in headers (green). Table from Gauto Benitez et al [18]. HFNC: high flow nasal cannula.

Tables and their descriptions typically contain at least partial representations of metrics and their measurement contexts. Context can be assigned to dimensions (rows and columns) of the table. For example, Figure 1 displays a “2×1” context, meaning that the rows of the table correspond to 2 nested pieces of context (arms nested in data elements) and the columns correspond to 1 piece of context (time points) [17]. For comparison, Figure 2 displays a “1×1” context, wherein the data elements are labeled on the rows (with corresponding statistical formats) and arms are defined in the columns (time points not presented) [18].

### Table Classification

#### Classification System

Each included record underwent full text review and was tagged in AutoLit based on the attributes of its (1) table structure, (2) measurement context, and (3) metric information. All tables within a published article were considered for classification via the tagging hierarchy. Tags were assigned on a per-table basis within each record such that the tags described the attributes of its tables.

#### Table Structure Reporting

As defined in Table 2, table attributes covered the reporting of baseline and outcome data and table orientation/pagination. If a table did not report any descriptive statistics concerning patient characteristics or outcomes, it was accordingly not tagged.

**Table 2:** Tagging hierarchy of table structural attributes.

Tags		Applied when
**Baseline or demographic characteristics of one or more study arms reported**		
	In table	The article reports baseline characteristics for the study population in a table, broken out by arm
	Participant level	The article reports baseline characteristics for the study population in a table, reported for each participant
	No arm-level breakout	The article reports baseline characteristics for the entire study population in a table, with no breakout
**Experimental outcomes reported**		
	In table	The article reports outcomes in a table, including the primary outcome(s), at one or more follow-up time points
	Participant level	The article reports outcomes in a table, including the primary outcome(s), at one or more follow-up time points, reported for each participant
	Secondary only, in table	The article reports outcomes in a table, but not all the primary outcomes of the study, instead focusing on data points other than primary outcomes
**Other table features**		
	Rotated 90 degrees	One or more baseline or outcome tables in the article is rotated 90 degrees in either direction on the page but is otherwise normal
	Multipage	One or more baseline or outcome tables in the article overflows beyond its starting page but is otherwise normal

#### Measurement Context Reporting

Measurement context tags were applied for all tables for which patient baseline characteristics or outcomes were reported. Contextual information of interest included the pieces of context per array (where an array represents either a row or column), including data element headers, arm names and arm size reporting, labeling of interventions, and labeling of time points (Table 3).

**Table 3:** Tagging hierarchy of measurement context attributes.

Tags			Applied when
**Alignment of table dimensions and measurement context^a^**			
	1×1	Only two pieces of context are shown in the table dimensions: one on rows and one on columns (eg, data elements on rows and arms columns)	
	2×1	Three pieces of context are shown in the table dimensions: two on rows and one on columns (eg, arms nested in data elements on rows, time points on columns)	
	1×2	Three pieces of context are shown in the table dimensions: one on rows and two on columns	
**Number of participants in individual arms of the study reported**			
	Embedded in arm	Arm sizes are reported as part of the arm or intervention label (eg, “Placebo [n=25]”)	
	Separate array	Arm sizes are reported in a distinct column or row in the table	
**Header labels corresponding to the intervention(s) applied in each arm of the study**			
	Full name	The entire name of the intervention(s) for the arm is shown in the arm header	
	Acronym or abbreviation	An acronym or shortened version of the invention name(s) for the arm is shown in the arm header	
	Control/experimental	The arm header is labeled with “Control” and “Experimental” or “Treatment” or “Intervention“	
	Alternate labels	Any header labeling scheme not identified above is used	
**Header labels corresponding to the time at which the reported data were collected**			
	Contains unit of time	The time point header contains an amount of time, including units	
	Pre/post	The time point header is labeled “Pre/Post,” “Before/After,” “Baseline/Follow-up”	
	Incremental numbered	Time point headers are labeled with numbers or letters in order of time (eg, “t_1_,” “t_2_”)	

^a^Context is tagged as “Embedded” when individual header cells include 2 elements of context (eg, “Baseline BMI”).

#### Metric Reporting

Unlike table structure and measurement context, metric tags were applied per article rather than per table. All baseline and outcome tables in the article were considered for metric classification (Table 4).

**Table 4:** Tagging hierarchy of metric attributes.

Tags		Applied when
**The format of statistics reported in a metric is displayed^a^**		
	In header	The statistic format or just constituents are reported in the header of the array of metrics
	In description or footnotes	The statistic format or constituents are reported in the description or footnotes; these may apply to the entire table or be annotations for arrays
**Units of continuous data elements reported^b^**		
	In header	The units of data elements are reported in each array header
	In descriptions or footnotes	The units of continuous data elements are reported in the description or footnotes; these may apply to the entire table or be annotations for arrays
	Not relevant	The article includes no continuous data elements or the continuous data elements are unitless (eg, scale data)
**Pattern defining how the constituent statistics in array of metrics are formatted^c^**		
	Continuous	The format is used for continuous data elements
	Dichotomous	The format is used for dichotomous data elements, specifically when only a single category is implied (eg, “Mortality” or “Gender Male”)
	Categorical	The format is used for categorical data elements; this also applies when a dichotomous data element explicitly lists all categories (eg, “Smoking” has separate arrays for “Yes” and “No”)
**Method of reporting category labels for categorical data elements^d^**		
	Separate array	Category labels are in an entirely separate (delimited) array from the data element header array
	Same array	Category labels are in the data element header array, with no distinction from other data element labels
	Same array indented	Category labels are in the data element header array, but are nested under the categorical data element header via white space or list indentation
	In cell	Categories are all reported in the same cell (eg, “Gender M/F” with metrics “11/9”)

^a^If multiple cases apply, the lowest in the table is the classification.

^b^If multiple cases apply, the lowest in the table is the classification. If units are missing on one or more data elements, this classification should be left empty.

^c^The formats under each tag are created as they are encountered in articles.

^d^This classification is left empty in the event that no categorical data elements are reported.

### Statistical Analysis

As a pilot study, no power analysis was performed to identify an appropriate sample size. Sample size was estimated to restrict 95% CIs on proportions to ±15% in width. Frequencies were compiled with Boolean queries on tags in AutoLit’s Study Inspector. Boolean queries were run on tagged articles using the open source software “btriev” [19] in NodeJS. The counts of results were then compiled and proportion CIs were generated using a normal approximation with the “prop.test” function in R. Inferential statistics on proportions were built with a normal approximation and computed in the R programming language. CIs are reported at the 95% level.

## Results

### Characteristics of Sampled Articles

**Figure 3:**
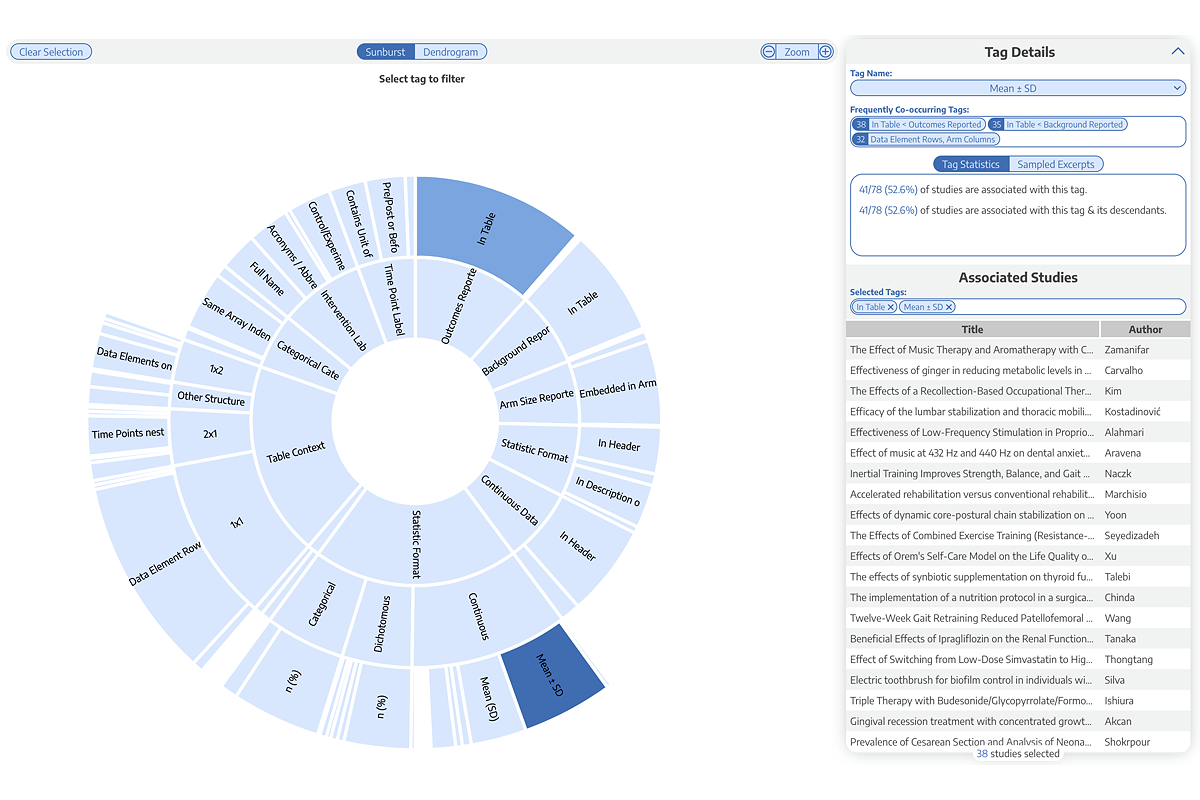
A screenshot of the interactive tagging hierarchy applied across the 78 studies included in this pilot survey. Two filters were applied: “Outcomes Reported In Table” was selected first, and then “Mean ± SD,” meaning the sunburst plot is filtered to studies for which both tags are present. The right menu displays the 38 studies for which this is true as well as statistics about how common the tags in question are across all included studies.

Of the sampled 100 records, 12 published articles were excluded for lack of PDF full text, 7 presented nonclinical findings, and 3 were not available in English, leaving 78 articles that were included in this pilot study. A single clinical study was reported in all (78/78, 100%) published articles. Articles were published in 65 distinct journals, with the most frequent journal, *PLoS One*, publishing 9 of the articles. An interactive visualization of all articles tagged using the hierarchical paradigm described above is available on the Nested Knowledge Synthesis page (see Figure 3) [20].

### Reporting of Baseline and Outcome Measures in Tables

Baseline and outcome data were reported in 71 of 78 (92%) articles (95% CI 84%-96%). Both baseline and outcome data were presented in tables in 66 of 78 articles (85%, 95% CI 75%-91%), and 77 (99%, 95% CI 93%-100%) articles reported at least one table of baseline or outcome measures. Standard reporting (with arm-level breakout) was present in 97% (64/66, 95% CI 90%-99%) of tables reporting baseline characteristics and in 96% (69/72, 95% CI 92%-99%) of tables reporting outcomes (Table 5).

**Table 5:** Baseline and outcome reporting per article and per table.

Type		Frequency per article (N=78), n (%)	Frequency per table (N=174), n (%)
**Baseline reported in table**		66 (85)	67 (38.5)
	Arm-level breakout	64 (97)	65 (97)
	No arm-level breakout	2 (3)	2 (3)
**Outcomes reported in table**		72 (92)	107 (61.5)
	Arm-level breakout	69 (96)	104 (97.2)
	Secondary only	2 (3)	2 (1.9)
	Participant level	1 (1)	1 (0.9)

Among the 174 tables that were found to report either baseline or outcome descriptive statistics, 6 (3.4%, 95% CI 1.6%-7.3%) were rotated 90 degrees and 5 (2.9%, 95% CI 1.2%-6.5%) were multipage.

### Reporting of Measurement Context in Tables

Table 6 shows the frequency of measurement contexts using the number of articles reporting one or more baseline or outcomes tables as the respective denominators. Overall, 48 (62%, 95% CI 51%-72%) articles labeled arms by the full intervention name or an abbreviation of it. Additionally, 22 of the 52 (42%, 95% CI 30%-56%) articles reporting the time point context in tables labeled time points according to the amount of time.

**Table 6:** Measurement context reporting per article (N=77).

Type		Frequency per relevant^a^ article, n (%)
**Arm size reported**		57 (74)
	Embedded in arm	50 (88)
	In separate array	6 (11)
	In description	1 (1)
**Intervention labels reported**		77 (100)
	Control/experimental	26 (34)
	Acronyms or abbreviated	25 (33)
	Full name	23 (30)
	Alternate labels	3 (4)
**Time point labels reported**		52 (68)
	Pre/post	24 (46)
	Contains unit of time	22 (42)
	Incremental numbering	6 (12)

^a^One article reported no baseline or outcome data in tables and was thus left out from the measurement context analysis.

In terms of dimensions, the 1×1 context of data elements and arms was most commonly used to report findings, with time points included in only 4 of 99 (4%, 95% CI 1.6%-9.9%) of these tables. Across all context types, arms were most frequently reported on columns (134/174, 77.0%), data elements on rows (144/174, 82.8%), and time points on columns (38/174, 21.8%) (see Table 7).

**Table 7:** Dimensions of tabular reporting of measurement context (N=174).

Context dimensions			Frequency per table, n (%)
**1×1**			99 (56.9)
	DEs^a^ on rows, arms on columns	90 (91)	
	Arms on rows, DEs on columns	5 (5)	
	Arm on rows, TP^b^ on columns	2 (2)	
	TPs on rows, arms on columns	2 (2)	
**2×1**			34 (19.5)
	TPs nested in DEs on rows, arms on columns	18 (53)	
	Arms nested in DEs on rows, TPs on columns	8 (24)	
	DEs nested in arms on rows, TPs on columns	2 (6)	
	Arms nested in TPs on rows, DEs on columns	2 (6)	
	DEs and TPs embedded in rows, arms on columns	2 (6)	
	TPs nested in arms on rows, DEs on columns	1 (3)	
	DEs nested in TPs on rows, arms on columns	1 (3)	
**1×2**			26 (14.9)
	DEs on rows, TPs nested in arms on columns	16 (62)	
	DEs on rows, arms nested in TPs on columns	5 (19)	
	Arms on rows, TPs nested in DEs on columns	3 (12)	
	DEs on rows, arms and TPs embedded on columns	2 (8)	
**Other structure**			15 (8.6)
	Stratified reporting	8 (53)	
	Only reports comparative statistics	7 (47)	

^a^DE: data element.

^b^TP: time point.

### Reporting of Metrics in Tables

Overall, 75 (97%) articles reported one or more continuous metrics in tables. Fourteen total formats were observed. Mean central tendencies were most frequently reported as mean ± SD (59%) and mean (SD) (36%), whereas medians were most commonly reported as median (25th percentile-75th percentile) (52%; see Table 8). Seven formats were used for means and 7 for medians.

Among 75 articles reporting at least one continuous metric, 64 (85%, 95% CI 76%-92%) contained data elements where units were relevant; of these, 55 (86%, 95% CI 75%-92%) reported units of measurement in table headers.

**Table 8:** Continuous metric reporting format in tables (N=77).

Continuous metrics reported		Frequency per relevant^a^ article, n (%)
**Mean**		69 (90)
	Mean ± SD	41 (59)
	Mean (SD)	25 (36)
	Mean and SD in separate arrays	2 (3)
	Mean	2 (3%)
	Mean SD	1 (1%)
	Mean (CI; lower-higher)	1 (1%)
	Mean ± SD (range; min-max)	1 (1%)
**Median**		21 (27)
	Median (IQR; 25th percentile-75th percentile)	11 (52)
	Median (range, min-max)	4 (19)
	Median [IQR; 25th percentile-75th percentile]	3 (14)
	Median [IQR; 25th percentile, 75th percentile]	1 (5)
	Median (IQR; 25th percentile to 75th percentile)	1 (5)
	Median (IQR)	1 (5)
	Min-Max (median)	1 (5)

^a^One article reported no baseline or outcome data in tables and was thus left out from continuous data characterization.

Regarding dichotomous and categorical metrics, 8 different formats were encountered across 62 articles (Table 9); the format n (%) was most commonly observed for both metric types.

Statistical formats (such as those shown in Table 9) were also commonly represented in tables. In 65 of 77 studies (84%), the generalizable format was given. In 35 articles, the format was presented in the header, whereas in 30 articles, it was presented in the footnote or tabular description.

**Table 9:** Dichotomous and categorical formats and labels in tables (N=77).

Statistics reported		Frequency per relevant^a^ articles, n (%)
**Dichotomous**		40 (52)
	n (%)	30 (75)
	n	3 (8)
	n/(N–n)	3 (8)
	%	3 (8)
	n, %	2 (5)
	n and % in separate arrays	1 (3)
**Categorical**		47 (61)
	n (%)	40 (85)
	n	8 (17)
**Categorical labels**		47 (61)
	Same array, indented	35 (74)
	Separate array	7 (15)
	In cell	7 (15)
	Same array, unindented	1 (2)

^a^One article reported no baseline or outcome data in tables and was thus left out from dichotomous/categorical data characterization.

Among the 47 articles reporting categorical metrics, category label indentation under the data element header was observed in 35 (74%, 95% CI 60%-85%) articles. Two articles used two distinct methods of category labeling.

Across all data types, statistic formats were reported in tables or their descriptions in 84% (95% CI 75%-91%) of articles.

### Characteristics of Articles With High Information Density

Notwithstanding the variety of measurement contexts (with 15 independent formats detected) and statistic formats presented, high information density was common in our dataset. Articles that were found to present maximal information in tables have the following classifications: baseline or outcomes reported in table; arm size reported; intervention labels should be full name or acronyms or abbreviations; when reporting relevant metrics, units of measurement are reported; and statistic format reported.

Thirty-one of the 78 sampled articles (40%, 95% CI 30%-51%) matched these classifications. The most impactful constraint among these classifications was “intervention labels”; if this classification is dropped, 48 (62%, 95% CI 50%-75%) articles matched the maximal-density, most-common formats listed above.

## Discussion

### Principal Results

In this pilot survey, we found that 85% (66/78) of articles reported both baseline and outcome data in tables, and 99% (77/78) of articles have at least one table of baseline or outcome data. Arm-level (intervention-specific) data were presented in 96% (69/72) of these tables, but there was major heterogeneity in the methods of reporting population size and intervention group names. Tabular dimensions ranged widely, with 15 independent dimensional structures used to report measurement context across 174 tables. Although 1×1 contexts represented the majority of tables, our results suggest that automated context detection will need to contend with a diversity of arrangements.

Similarly, although continuous data were very commonly reported (90% of articles, n=69/77) and dichotomous and categorical data were consistently reported (52% and 61% of articles, n=40/77 and n=47/77 respectively), statistic formats were heterogeneous, with seven formats for means, seven for medians, and six for dichotomous data. Despite the heterogeneity of format, tables provided a consistent, high-density source for baseline and outcome data, and the contexts and formats defined here can be used to refine the expected data presentation for tabular data extraction. We plan to expand upon this pilot study in tabular structure with a review-and-tabular-extraction study, wherein the framework outlined here will be used to classify and extract from underlying articles and the accuracy of extracted metrics will be determined by comparison to manual extraction.

### Automated Recognition of Context and Format

The context presented in tables showed the most disappointing rate of reporting information of interest. Although arm sizes were reported in tables in 74% (57/77) of publications, arm interventions and measurement time points were reported in a self-contained manner 60% (46/77) and 40% (31/77) of the time, respectively. If tables are extracted in a completely self-contained manner, with no access to the publication’s full text, we expect only 40% of publications will contain sufficient information in tables alone to complete extraction. Extraction automations will therefore have to receive human input or consult the free text to supplement arm and time point contexts from the tables.

Statistic reporting was heterogenous in format but extremely commonly reported: statistic format was explicitly reported in tables in up to 90% (69/77) of publications. Even where absent, the format may also be inferred from the metric arrays themselves. Inferred formats may be useful when formats are not reported or as a validation on the detected format. Categorical metrics may produce the most complexity for automations, as category labels are often not distinguished from data element labels by more than whitespace indentation.

Given the commonality of n (%) reporting for dichotomous and categorical data elements, it may be possible to arithmetically derive missing arm sizes from metrics. If not essential, mining the full text may still provide value in validations or completing data. For example, interventions were reported as abbreviations or acronyms in around 30% (23/77) of publications; pattern matching on the abstract or introduction could generate full strings for these shortened versions.

A nontrivial number of publications, around 5% (4/77), contained rotated or multipage tables. Automations should consider tools to identify and apply corrective measures in these cases. Eight percent of tables reported only stratified data or only comparative statistics; although these cases are typically mathematically correctable to arm-level data that meta-analysts desire, automation of these procedures would add complexity. Lastly, although no publication included in our pilot study had missing data, potential missing data must be addressed in any automated workflow: we suggest that for any table where data are missing, the table should be visually presented to users for confirmation.

### Previous Research on Tabular Extraction

To date, the scientific literature does not seem to contain any studies giving an overview of the table structure, context presentation, and statistic formats, making this pilot study the first of its kind. The Cochrane Collaboration has created a test data set for automated extraction that may be used to test the accuracy of novel extraction algorithms; however, their data set did not classify tabular structure, instead focusing on providing the test/training data sets and preliminary testing of their own semiautomated extraction system [21].

However, previous authors have proceeded beyond classification and provided approaches for automating tabular extraction. Unlike the approaches reviewed by Jonnalagadda et al [8] and Marshall et al [22], which focus on simplified content-extraction tasks from free text (such as abstracts), Milosevic et al [23] actually tested a preliminary algorithm for tabular extraction. Although this study did not include an overview of the context or statistic formats, the authors achieved an F-score (accuracy) of 82%-92% in the content extraction from a simplified set of HTML tables with 1×1 context formats. The seven-step process for detecting, processing, tagging, and extracting from tables used by Milosevic et al [23] is the most complete tabular extraction process published to date. The only competing approach focusing on tables in medical publications was from Xu et al [24], who were able to extract drug side effect relationships with 52% accuracy using a statistical classifier. Other than Milosevic et al’s [23] pilot study, despite at least 26 approaches attempted in textual extraction [8], automated extraction remains an unmet need for which tabular extraction is a promising and underexamined methodology.

### Limitations

We believe our findings will generalize to modern clinical publications owing to the simplicity of our search and applicability of classifications. However, this survey and the AutoLit data extraction framework are applicable only to clinical research publications. Since our search was limited to RCTs, some study types such as observational studies may show different characteristics. Similarly, we did not stratify our results by journal, impact factor, or other factors apart from filtering to RCTs, although journal-related characteristics may affect how representative this pilot is of medical publishing generally.

Additionally, specific fields of research may show characteristics that do not align well with the averaging-across-fields approach used in our study. As a pilot survey, our study did not involve a power analysis; however, this pilot study can be used to determine sample sizes quantitatively in future research. Lastly, our breakout of contexts and formats is always subject to expansion not covered in this sample, and automations built on the expectation of a limited set of contexts or formats may fail when new presentations of this information are encountered. The test of this framework will be the accuracy of extraction algorithms that employ it compared against existing extraction methods.

### Conclusions

In this pilot survey, we found a high density of information in tables, with over 85% (66/78) of articles reporting both background and outcome measures in tables, but with major heterogeneity in presentation of measurement context. Measurement context was most often presented in a 1×1 format, but 15 independent formats were found. Similarly, means and medians were each found in seven independent formats, and dichotomous variables in six. Despite this, high-quality contextual information (intervention labels, arm sizes, units, and statistic formats) were presented in 40% (31/77) of articles. The range of context and statistic formats surveyed here can provide a baseline for future tabular extraction efforts.

## Abbreviations

RCT: randomized controlled trial
